# Targeting cGAS-STING: modulating the immune landscape of hepatic diseases

**DOI:** 10.3389/fimmu.2025.1498323

**Published:** 2025-03-03

**Authors:** Feng Xue, Yong-Kang Liu, Xiao-Ying Chen, Shan-Shan Chen, Xiang-Rong Yu, Hua-Wen Li, Li-Gong Lu, Mu-He Chen

**Affiliations:** ^1^ Department of Radiology, Zhuhai Clinical Medical College of Jinan University (Zhuhai People's Hospital, The Affiliated Hospital of Beijing Institute of Technology), Zhuhai, Guangdong, China; ^2^ Guangdong Provincial Key Laboratory of Tumor Interventional Diagnosis and Treatment, Zhuhai Institute of Translational Medicine, Zhuhai, Guangdong, China; ^3^ Guangzhou First People's Hospital, the Second Affiliated Hospital, School of Medicine, South China University of Technology, Guangzhou, China; ^4^ Institute of Translational Medicine, Zhuhai Clinical Medical College of Jinan University (Zhuhai People's Hospital, The Affiliated Hospital of Beijing Institute of Technology), Zhuhai, Guangdong, China; ^5^ Department of Gynecology, Zhuhai Clinical Medical College of Jinan University (Zhuhai People's Hospital, The Affiliated Hospital of Beijing Institute of Technology), Zhuhai, Guangdong, China

**Keywords:** cGAS-STING, tumor immune microenvironment, hepatocellular carcinoma, viral hepatitis, alcoholic liver disease, metabolic dysfunction-associated steatotic liver disease

## Abstract

Liver diseases, including viral hepatitis, alcoholic liver disease (ALD), metabolic dysfunction-associated steatotic liver disease (MASLD), and hepatocellular carcinoma (HCC), represent a significant threat to global health due to their high mortality rates. The cGAS-STING pathway, a critical part of the innate immune system, plays a crucial role in detecting cytoplasmic DNA and initiating immune responses, including autoimmune inflammation and antitumor immunity. Genomic instability during cancer progression can trigger this pathway by releasing DNA into the cytoplasm. Emerging research indicates that cGAS-STING signaling is intricately involved in maintaining liver homeostasis and contributes to the pathogenesis of various liver diseases. This review outlines the cGAS-STING pathway, with a particular focus on its activation mechanism and its roles in several notable liver conditions. Specifically, we explore the complex interplay of cGAS-STING signaling in viral hepatitis, ALD, MASLD, and HCC, and discuss its potential as a therapeutic target. For example, in HCC, strategies targeting cGAS-STING include using nanomaterials to deliver STING agonists, combining radiofrequency ablation (RFA) with cGAS-STING activation, and leveraging radiotherapy to enhance pathway activation. Furthermore, modulating cGAS-STING activity may offer therapeutic avenues for viral hepatitis and chronic liver diseases like MASLD and ALD, either by boosting antiviral responses or mitigating inflammation. This review highlights the complex role of cGAS-STING signaling in these specific liver diseases and underscores the need for further research to fully realize its therapeutic potential.

## 1 Introduction

Liver diseases encompass a spectrum of conditions, including but not limited to viral hepatitis, metabolic dysfunction-associated steatotic liver disease (MASLD, previously termed NAFLD), alcoholic liver disease (ALD), and hepatocellular carcinoma (HCC). Cellular oxidative stress and inflammatory responses are key drivers in the pathogenesis of many liver diseases, leading to excessive production of extracellular matrix. Excessive buildup of extracellular matrix initiates a cascade of detrimental effects on the liver, starting with structural disruption and functional impairment, and progresses to fibrosis, cirrhosis, and ultimately HCC (1). The global burden of liver diseases is substantial, with these conditions being responsible for a significant proportion of mortality globally and representing a major public health challenge. They are estimated to be the 11th most common cause of death on a global scale (2). HCC alone is responsible for a substantial portion of these deaths, claiming between 600,000 to 900,000 lives annually (2). Clinical management of liver diseases varies depending on the underlying etiology and disease stage. Pharmacotherapy plays a crucial role in managing conditions such as viral hepatitis and MASLD. Surgical interventions, including resection and transplantation, are often considered for early-stage HCC and certain other liver conditions. However, because early stages of HCC often lack noticeable symptoms, the disease frequently progresses undetected until a late stage, resulting in a poor prognosis (3). Immune checkpoint inhibitor (ICI) based combination therapies have shown promising results in treating HCC, as demonstrated by the IMbrave150 trial and other Phase 3 trials, marking a significant advancement in immunotherapy for liver cancer (4–7). While ICIs have demonstrated efficacy in a subset of HCC patients, a significant proportion of patients do not experience durable responses. Tumor heterogeneity, immune evasion, and treatment resistance limit the efficacy of current therapies. Consequently, research efforts are directed towards identifying predictive biomarkers and developing innovative combination therapies to improve treatment response and overcome these hurdles (8).

The cyclic GMP-AMP synthase (cGAS)-stimulator of interferon genes (STING) pathway is a central component of the innate immune response to cytoplasmic DNA. Functioning within the cytoplasm, cGAS detects anomalous double-stranded DNA (dsDNA), which can originate from a variety of sources such as viral infection, cellular stress, and genomic instability (9–11). dsDNA binding induces a conformational shift in the C-terminal domain of cGAS, activating its catalytic function and producing the signaling molecule 2’3’-cGAMP using ATP and GTP. The cyclic dinucleotide 2’3’-cGAMP, formed by adenosine and guanosine linked via phosphodiester bonds, acts as an intracellular second messenger, activating STING upon binding. The N-terminal domain of cGAS is involved in DNA binding and contributes to the formation of cGAS-DNA complexes (12). As a homodimer, the transmembrane protein STING resides within the endoplasmic reticulum (ER) membrane. The human STING (hSTING) protein is structured with a trans-membrane domain at its N-terminus featuring four helices, an acidic tail at the C-terminus, and a central, spherical domain that divides the two aforementioned regions (13). In liver tissue, hepatocytes and liver cancer cells do not express STING protein, and stimulating the cGAS-STING pathway mainly relies on non-parenchymal liver cells (14). Upon detecting cytoplasmic dsDNA, which can indicate cellular damage or pathogen presence, cGAS activates the downstream STING protein through cGAMP and initiates a series of cascade reactions, ultimately promoting the expression of Type I Interferon (INF-I), initiating autoinflammatory responses, antiviral, and tumor suppression functions (13).

The discovery of the role of cGAS-STING pathway in liver disease offers exciting new avenues for therapeutic intervention, potentially leading to novel treatments. This article presents an in-depth exploration of the cGAS-STING pathway, with a particular emphasis on its activation process and its multifaceted implications for liver disease. Our initial focus is on the role of cGAS-STING pathway in HCC tumor suppression, exploring how it modifies the tumor immunological environment and ultimately leads to cancer cell clearance. Secondly, we will delve into how the cGAS-STING pathway is implicated in various liver conditions, including viral hepatitis, MASLD, and ALD, exploring its diverse functions in each. Lastly, we will address potential avenues for future research and the translational potential of manipulating cGAS-STING signaling as a therapeutic strategy for liver pathologies.

## 2 The cGAS-STING signaling pathway

**Figure 1:**
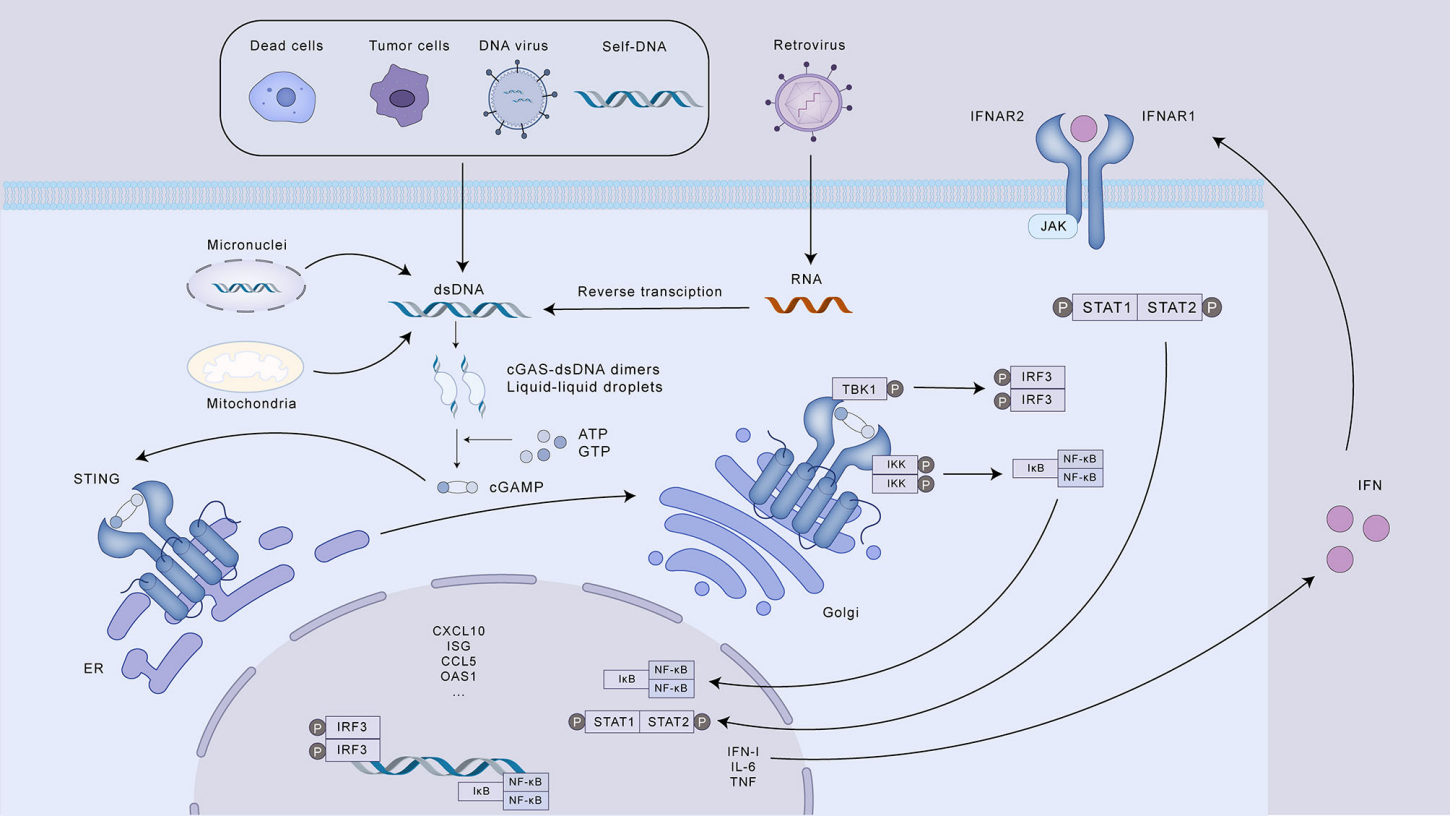
The cGAS-STING signaling pathway and its role in innate immunity. This illustration depicts the activation and downstream signaling cascade of the cGAS-STING pathway, a crucial component of the innate immune response to cytosolic DNA. Various sources, including dead cells, tumor cells, DNA and retroviruses, and self-DNA, can release dsDNA into the cytoplasm, triggering cGAS activation. Upon binding to dsDNA, cGAS catalyzes the synthesis of cGAMP, which acts as a second messenger to activate STING on the ER. Activated STING translocates to the Golgi apparatus, where it activates TBK1, leading to the phosphorylation of both STING and IRF3. Phosphorylated IRF3 dimerizes and induces the transcription of IFN-I. Simultaneously, STING activates IKK, resulting in the release and nuclear translocation of NF-κB, which promotes the transcription of pro-inflammatory cytokines. IFN-I, in turn, binds to its receptor (IFNAR) and initiates the JAK-STAT signaling pathway, leading to the expression of ISGs. These ISGs contribute to diverse immune responses, including enhanced antigen presentation, T cell and NK cell activation, antibody production, and direct pathogen elimination. The cGAS-STING pathway also regulates the inflammatory response through autophagy, which can degrade STING.

Typically, DNA resides within the cellular compartments of the nucleus and mitochondria. Upon disruption of cellular homeostasis and the subsequent accumulation of aberrant dsDNA in the cytosol, cGAS undergoes activation. Studies have shown that chromosomal instability (CIN) in cancer cells is a major source of dsDNA in the cytoplasm. Cancer cells characterized by genomic instability are prone to errors in chromosome segregation during mitosis, leading to the development of micronuclei, serving as storage compartments for chromosomal DNA in a cell cycle-regulated manner (15). The envelope of micronuclei is prone to rupture, exposing their genomes in the cytosol and triggering the cGAS-STING pathway (16, 17). In addition to dsDNA from micronuclei, dysfunctional mitochondria in tumor cells under oxidative stress may also be another source of genomic DNA (18). When cGAS senses misplaced dsDNA in the cytosol, it binds to the dsDNA in a non-sequence-specific 2:2 ratio, causing a conformational change in cGAS to form a cGAS-dsDNA dimer, and the activated cGAS catalyzes ATP and GTP into cGAMP. cGAMP, as an intracellular second messenger, has a high affinity for STING. cGAMP activates STING residing in the ER, prompting its trafficking to the Golgi complex through the ER-Golgi intermediate compartment. Within the Golgi apparatus, STING promotes the assembly and enzymatic activity of TBK1. This kinase subsequently phosphorylates STING, as well as the interferon regulatory factor 3 (IRF3). Following phosphorylation mediated by TBK1, STING undergoes autophagic degradation, which serves as a regulatory mechanism to prevent excessive innate immune responses. Concurrently, phosphorylated IRF3 undergoes dimerization and nuclear translocation, where it initiates the transcriptional activation of IFN-I genes. In addition, STING can also recruit IκB kinase (IKK), leading to the inactivation of IκB through phosphorylation. This allows NF-κB to translocate to the nucleus and initiate the transcription of genes encoding pro-inflammatory cytokines. IFN-I can stimulate Janus kinase 1 (JAK1) by interacting with its cognate receptor, the interferon receptor IFNAR1/IFNAR2, located on the cell surface. This interaction leads to the phosphorylation of STAT proteins (signal transducers and activators of transcription) and triggers the expression of a range of interferon-stimulated genes (ISGs). These ISGs encompass various effector molecules, including cytokines and chemokines, which can directly engage and eliminate pathogens, thereby controlling infections caused by viruses, bacteria, and parasites. Furthermore, IFN-I activates IFN-I receptors on other immune cells, amplifying the antiviral response. This includes: T cell and NK cell-mediated lysis of infected cells; B cell production of neutralizing antibodies; and increased IFN-I secretion by antigen presenting cells (APCs) to further propagate the immune response. Moreover, studies have demonstrated that activation of the cGAS-STING pathway can trigger both apoptosis and autophagy (19, 20). In the context of cGAS-STING signaling, autophagy plays a complex role by influencing inflammatory responses. This duality is achieved through both the induction of autophagy pathways (dependent or independent of TBK1) for the removal of harmful signals, and the targeted degradation of STING, a crucial component of the inflammatory cascade (**Figure 1**).

## 3 The immune microenvironment of HCC

**Figure 2:**
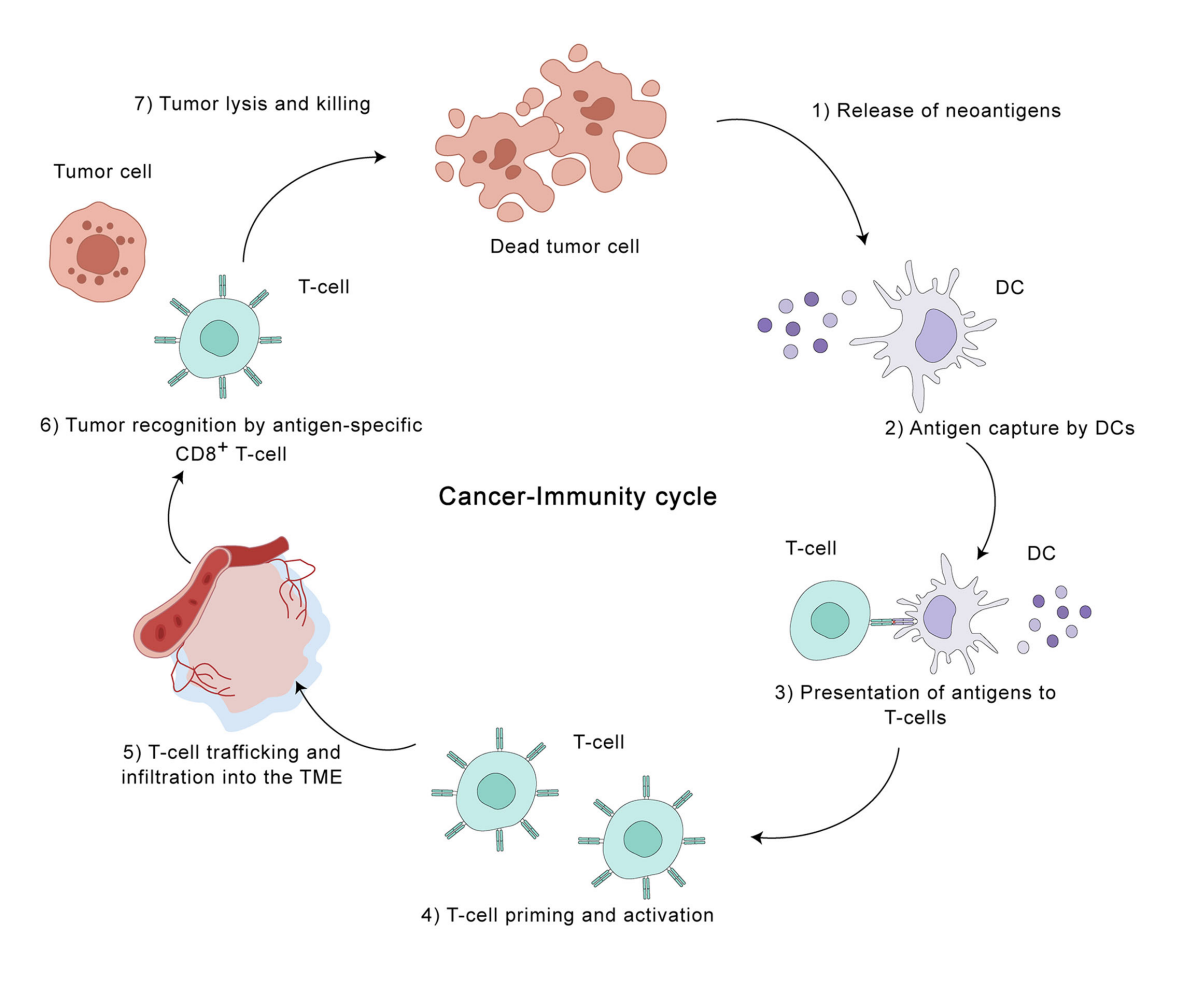
The cancer-immunity cycle in the hepatocellular carcinoma (HCC) tumor microenvironment. This illustration depicts the seven key steps involved in the cancer-immunity cycle, highlighting the dynamic interplay between tumor cells and the immune system within the HCC TME. The cycle is initiated by the release of tumor-specific antigens (neoantigens) from dying tumor cells (step 1). Dendritic cells (DCs) capture these antigens and migrate to lymph nodes, where they present them to T cells (steps 2 and 3). This presentation activates and primes antigen-specific CD8+ T cells (step 4), which then traffic to and infiltrate the TME (step 5). Within the TME, these activated T cells recognize and bind to tumor cells displaying the corresponding antigens (step 6), leading to tumor cell destruction (step 7). This cycle represents a critical process in anti-tumor immunity, but can be disrupted by various mechanisms employed by tumor cells to evade immune surveillance. Understanding the intricacies of the cancer-immunity cycle in HCC is crucial for developing effective immunotherapeutic strategies.

The HCC tumor microenvironment (TME) is a heterogeneous landscape encompassing not only malignant hepatocytes but also various immune cell subsets, a rich milieu of cytokines, and the structural framework of the extracellular matrix, and it exerts a considerable influence on disease progression (21). This intricate and ever-changing inter-tumoral immune landscape can be understood through the framework of the seven-step immunoediting process: 1. Neoantigen liberation: Tumor cells undergoing apoptosis or necrosis release tumor-specific antigens, frequently termed neoantigens. 2. Antigen Presentation: Dendritic cells (DCs) capture these neoantigens and subsequently present them within the lymph nodes. 3. T Cell Activation: This antigen presentation triggers the activation and subsequent differentiation of naive T cells into effector T cells. 4. Immune Cell Trafficking: Primed T cells subsequently undergo a process of directed movement to reach the tumor. 5. Tumor Infiltration: These activated T cells infiltrate the tumor microenvironment. 6. Cancer Cell Recognition: Inside the tumor, T cells engage with and destroy cancer cells displaying the corresponding antigens. 7. Cancer Cell Elimination: Finally, the activated T cells initiate a cytotoxic response, leading to the destruction of the recognized cancer cells (22) (**Figure 2**). The immune cells in the TME of HCC can be broadly categorized into two types: pro-tumor immune cells and anti-tumor immune cells. Pro-tumor immune cells, including regulatory T cells (Treg), myeloid-derived suppressor cells (MDSCs), and M2-polarized macrophages, can suppress effective immune responses against tumor cells and create an immunosuppressive tumor microenvironment; anti-tumor immune cells, including CD8 T cells, NK cells, DCs, and M1-polarized macrophages, can promote effective anti-tumor immune responses (23). Typically, a complex interplay among various cell types governs immune reactions, ensuring a balance between homeostasis and self-tolerance within the body. However, in the TME of HCC, the balance of these immune cell subsets is disrupted, leading to an immunosuppressive environment that favors tumor growth. Research indicates that reciprocal interactions between neoplastic cells and constituents of the immune milieu promote tumor immunoevasion (24).

Tumors can disrupt the cancer-immunity cycle and evade immune surveillance through intrinsic mechanisms (acquiring genetic mutations) or extrinsic mechanisms (producing an immunosuppressive TME) (22). Intrinsic mechanisms involve alterations within the tumor cells themselves, hindering their ability to be detected and destroyed by the immune system. These include impairments in the cellular machinery responsible for antigen processing and presentation, downregulation or absence of molecules known as major histocompatibility complex class I (MHC-I), thus preventing the presentation of tumor-associated antigens (TAAs) to cytotoxic T lymphocytes (CTLs), and epigenetic modifications that lead to the silencing of TAAs expression (25). The possible causes of extrinsic mechanisms of immune evasion in tumors are: 1) The presence of immunosuppressive cells, such as Tregs, MDSCs, and M2 macrophages, which actively suppress anti-tumor immune responses; 2) Co-inhibitory signals on lymphocytes, such as PD-1 and CTLA-4, which are upregulated in the TME and suppress T cell activation and function; 3) Cytokines such as TGF-β and IL-10, along with other soluble factors and signaling pathways, dampen immune cell activity and foster tumor growth. 4) A metabolically unfavorable tumor microenvironment characterized by hypoxia and nutrient deprivation, which hinders the infiltration and function of immune cells; 5) Tumor microbiome dysbiosis can remodel the immune microenvironment, promoting the advancement of HCC (26–31).

## 4 Impact of cGAS-STING pathway activation on the tumor microenvironment: from “cold” to “hot” tumors

**Figure 3:**
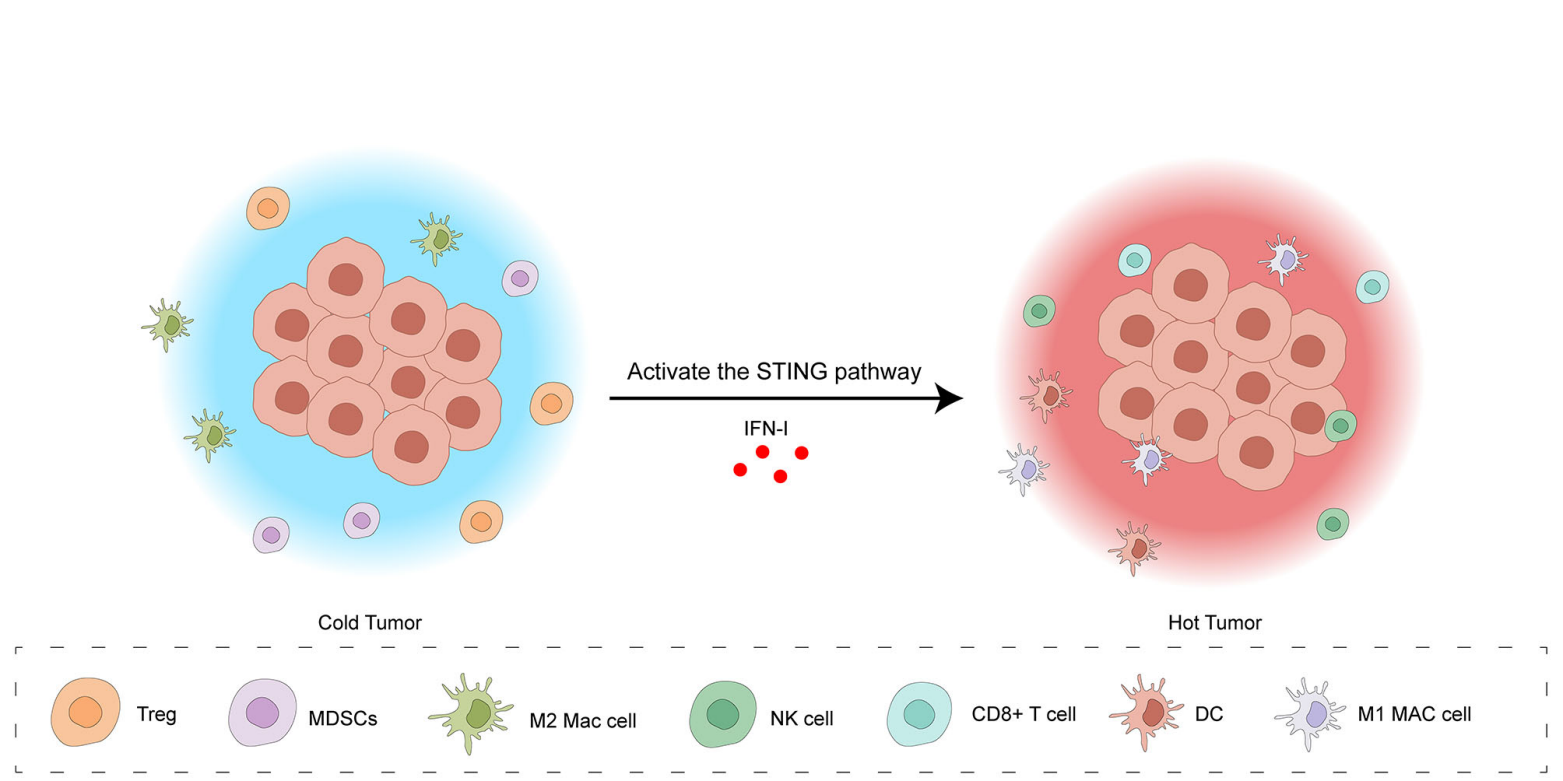
Activation of the cGAS-STING pathway transforms the tumor microenvironment from a “cold” to a “hot” state. The “cold” tumor microenvironment is characterized by a low density of immune cells, including Tregs, MDSCs, M2 macrophages, and NK cells, resulting in limited anti-tumor immunity. Activation of the STING pathway, triggered by IFN-I, induces a shift to a “hot” tumor microenvironment. This “hot” state features an influx of cytotoxic immune cells, such as CD8+ T cells, DCs, and M1 macrophages, indicating a robust anti-tumor immune response. This transition is facilitated by IFN-I-mediated enhancement of tumor cell immunogenicity, recruitment of effector immune cells, and suppression of immunosuppressive cell activity.

The location of CD8+ CTLs within the TME is a key factor in categorizing tumors into three distinct immunophenotypes: inflammatory, exclusionary, and desert-like. Inflammatory tumors, also termed “hot” tumors, are defined by an elevated tumor mutation load, heightened pro-inflammatory signaling pathways (predominantly driven by interferon-γ), increased expression of immune checkpoint proteins, such as PD-L1, and a pronounced infiltration of CTLs. These features often, but not always, correlate with a better response to ICIs therapy. In contrast, exclusionary and desert-like tumors, termed “cold” tumors, are characterized by a lower tumor mutation load, reduced expression of MHC-I, downregulation of the immune checkpoint molecule PD-L1, and a decreased infiltration of CTLs. Furthermore, immunosuppressive cells, including MDSCs, tumors-associated macrophages (TAMs), and Tregs, actively contribute to immune evasion in cold tumors. In exclusionary tumors, T cells are often found in the periphery of the tumor but are unable to infiltrate the tumor core. In contrast, desert-like tumors lack significant T cell infiltration altogether (32). (**Figure 3**) As discussed, the cGAS-STING axis activation triggers IFN-I production, a critical factor in fostering anti-cancer immunity (33). Specifically, IFN-I enhances tumor immunogenicity and facilitates the transition of “cold” tumors to an inflamed “hot” phenotype, marked by improved responsiveness to ICIs and increased T cell infiltration (32). IFN-I engages with IFNAR1/IFNAR2 receptors on diverse immune cells within the TME, initiating a series of intracellular processes that collectively remodel the TME, promoting an environment conducive to anti-tumor immunity. In tumor cells, IFN-I enhances MHC-I and TAAs presentation, augmenting TAAs recognition and processing by APCs, such as DCs, for subsequent presentation to CD8+ T lymphocytes (34, 35). Additionally, it can trigger tumor cell death through apoptosis and impede both their proliferative capacity and metastatic potential (36). Some research indicates that IFN-I can promote the development and functional maturation of both DCs and NK cells. This leads to an augmentation of NK cell cytotoxicity and fosters the trafficking of DCs to the tumor site (37). However, the function of IFN-I on NK cells remains controversial. For example, Amanda J.’s research showed that type I IFN can inhibit the immune function of NK cells during HSV-2 infection and cytokine stimulation (38). Within the Treg cells population, IFN-I-mediated signaling results in the production of IL-6, an cytokine that drives the transdifferentiation of Tregs into Th17 cells (37). Concurrently, IFN-I can indirectly suppress Treg trafficking to the TME through the inhibition of CCL22-mediated chemotaxis (39). In MDSCs, the application of IFN-I interferes with their developmental and maturation processes, thereby diminishing their suppressive influence on T cell expansion (40). Professor Li’s research shows that hyperbaric oxygen (HPO) can promote the teniposide-induced activation of the cGAS-STING signaling pathway, enhancing the anti-tumor efficacy of PD-1 antibodies in HCC. Teniposide, a DNA topoisomerase II inhibitor, activates the cGAS-STING pathway by inducing DNA breakage and damage in human HCC cells. Given that HCC is a solid tumor characterized by severe TME hypoxia, however, the teniposide-induced activation of the cGAS-STING pathway is significantly inhibited by hypoxia-inducible factor 1α (HIF-1α), thus contributing to a “cold” tumor. Conversely, the combined administration of hyperbaric oxygen and teniposide can abrogate the inhibitory effect of HIF-1α, thereby promoting a “hot” tumor (41).

Stimulating the cGAS-STING pathway can affect the occurrence and development of HCC and reduce the tumor burden in advanced HCC. The research of Thomsen, utilizing a drug-induced HCC mouse model, revealed that the absence of STING correlated with a marked elevation in tumor burden as the liver cancer progressed. Then, cyclic dinucleotides (CDN) was used to treat STING-deficient mice and found that it could promote tumor cell apoptosis, autophagy, and produce an IFN-I response, reducing the tumor and alleviating the tumor burden (42). In HCC, macrophages, such as Kupffer cells (KCs), can be activated by the cGAS-STING pathway when a large amount of DNA is released from dying tumor cells or when exogenous STING agonists are used. Activated KCs exert anti-tumor effects primarily through two distinct processes. Firstly, they augment the presentation of tumor-associated antigens, thereby triggering an adaptive immune response. Secondly, they secrete cytokines, including TNF-α and IL-12, which subsequently attract and activate CTLs to the tumor microenvironment (42).

In summary, engaging the cGAS-STING pathway within the TME exerts a multifaceted anti-tumor effect. Firstly, this process initiates the synthesis of IFN-I, a cytokine essential for the maturation and activation of APCs. Consequently, this facilitates the display of tumor antigens and triggers the development of cytotoxic CD8+ T cell responses directed against the tumor. Furthermore, IFN-I directly bolsters both the proliferative capacity and cytotoxic functionality of CD8+ T cells. Secondly, activation of the cGAS-STING pathway leads to the release of chemokines, attracting effector T cells to the TME and ultimately enhancing the destruction of tumor cells. This culminates in the conversion of a “cold” tumor microenvironment, characterized by limited immune infiltration, to a “hot” tumor microenvironment, rich in cytotoxic immune cells.

## 5 cGAS-STING pathway: a novel therapeutic target for HCC

### 5.1 Activating cGAS-STING with nanomaterials: promising strategies for HCC immunotherapy

Due to the ability of the activated cGAS-STING pathway to elicit anti-tumor immunity in HCC, targeted and effective activation of this pathway within HCC tissue has become a critical research priority. 5,6-dimethylxanthenone-4-acetic acid (DMXAA) is a vascular disrupting agent that inhibits angiogenesis by reducing VEGF levels. DMXAA can also activate the STING pathway in mice and has shown promising antitumor effects in murine models (42). A study investigated the use of a metal-organic framework, MOF-801, as a delivery vehicle for the STING agonist DMXAA and CpG ODNs for the treatment of HCC. The resulting nanoparticle, MOF-CpG-DMXAA, effectively delivered DMXAA and CpG ODNs to tumor cells, leading to a synergistic improvement in TME. This was achieved by reprogramming TAMs to an M1 phenotype, promoting DCs maturation, and destroying tumor blood vessels. In mouse models, MOF-CpG-DMXAA triggered robust anti-tumor immunity and significantly inhibited tumor growth and recurrence. Interestingly, the study also found that MOF-801 itself can act as a STING agonist through TLR4 recognition, further contributing to the overall therapeutic efficacy (43). It is important to note that DMXAA has been proven ineffective in activating human STING protein (44). A new type of STING agonist, MSA-2, has been developed recently, which can effectively induce IFN-I in mice and humans and has shown significant efficacy in promoting tumor regression (45). To address the potential for unnecessary inflammation in normal tissues caused by systemic administration of MSA-2, Feng et al. designed and synthesized an injectable hydrogel system, ALG@MSA-2, which is locally injected into residual HCC tissue after RFA treatment of HCC (45). The sustained release of MSA-2 within the residual HCC tissue triggers the cGAS-STING cascade. This localized activation counteracts the immunosuppressive environment induced by incomplete RFA, ultimately promoting an antitumor immune response. Unlike the direct activation of the pathway using STING agonists, Li’s team designed and synthesized a gold complex targeting liver mitochondria. By disrupting mitochondrial function in HCC cells, this mechanism results in the buildup of reactive oxygen species (ROS), leading to oxidative stress. This, in turn, triggers endoplasmic reticulum stress (ERS), ultimately culminating in immunogenic cell death (ICD). In addition, mitochondrial DNA (mtDNA) leaked into the cytosol can be recognized by cGAS, leading to the activation of the cGAS-STING pathway (46). Du et al. designed mesoporous silica nanoparticles loaded with Fe and Mn. These nanoparticles target HCC tissue. The Fe component triggers pyroptotic cell death, leading to the release of dsDNA, which subsequently initiates the activation of the cGAS-STING pathway (47). The Mn component enhances the sensitivity of cGAS, amplifying the activation of the pathway (48).

### 5.2 Boosting antitumor immunity in HCC: combining radiofrequency ablation with cGAS-STING activation

As a prevalent treatment for HCC, RFA offers a minimally invasive approach to eliminate tumors through thermal destruction, while also inducing the release of numerous TAAs (49). RFA significantly alters the HCC immune microenvironment, leading to a shift in macrophage populations toward an M2-dominant profile and impairing the antigen-presenting capacity of dendritic cells. These changes ultimately contribute to the establishment of an immunosuppressive milieu (45). Zhou’s team designed and synthesized an *in situ* nano-vaccine (LDHs-cGAMP) composed of layered double hydroxides carrying cGAMP and capable of adsorbing TAAs. After the completion of RFA, LDHs-cGAMP is injected locally, and it adsorbs the TAAs released by RFA. Following uptake by cancer cells and immune cells alike, LDHs-cGAMP-TAAs initiate the cGAS-STING signaling cascade, resulting in the generation of IFN-I, CXCL10, and other molecules that enhance CTL infiltration into the tumor. Furthermore, combining this approach with αPD-L1 antibody therapy significantly improves treatment response, ultimately generating a robust anti-tumor immune response (49). OK-432 is a preparation made from penicillin-killed and freeze-dried pyogenic streptococcus (Su), a strain of hemolytic streptococcus group A (Streptococcus pyogenes), and has served as an immunomodulator in the treatment of diverse cancers (50). Sun et al. investigated the synergistic effect of combining RFA and OK-432 in HCC treatment, discovering that OK-432 stimulates the cGAS-STING pathway in DCs, an effect further amplified by RFA (51). Cao et al. revealed that lysed OK-432 (lyOK-432) improves DC recruitment and maturation due to its ability to deliver a greater quantity of exogenous antigens and bacterial DNA rich in unmethylated CpG motifs. The application of a lyOK-432 and doxorubicin-loaded RADA16-I hydrogel to residual HCC tissue after RFA promotes DC recruitment and maturation within the tumor. This intracellular stimulation of the cGAS-STING pathway culminates in an enhanced anti-tumor immune response (50).

### 5.3 Exploiting the DNA damage response: harnessing radiotherapy to activate the cGAS-STING pathway in HCC

**Figure 4:**
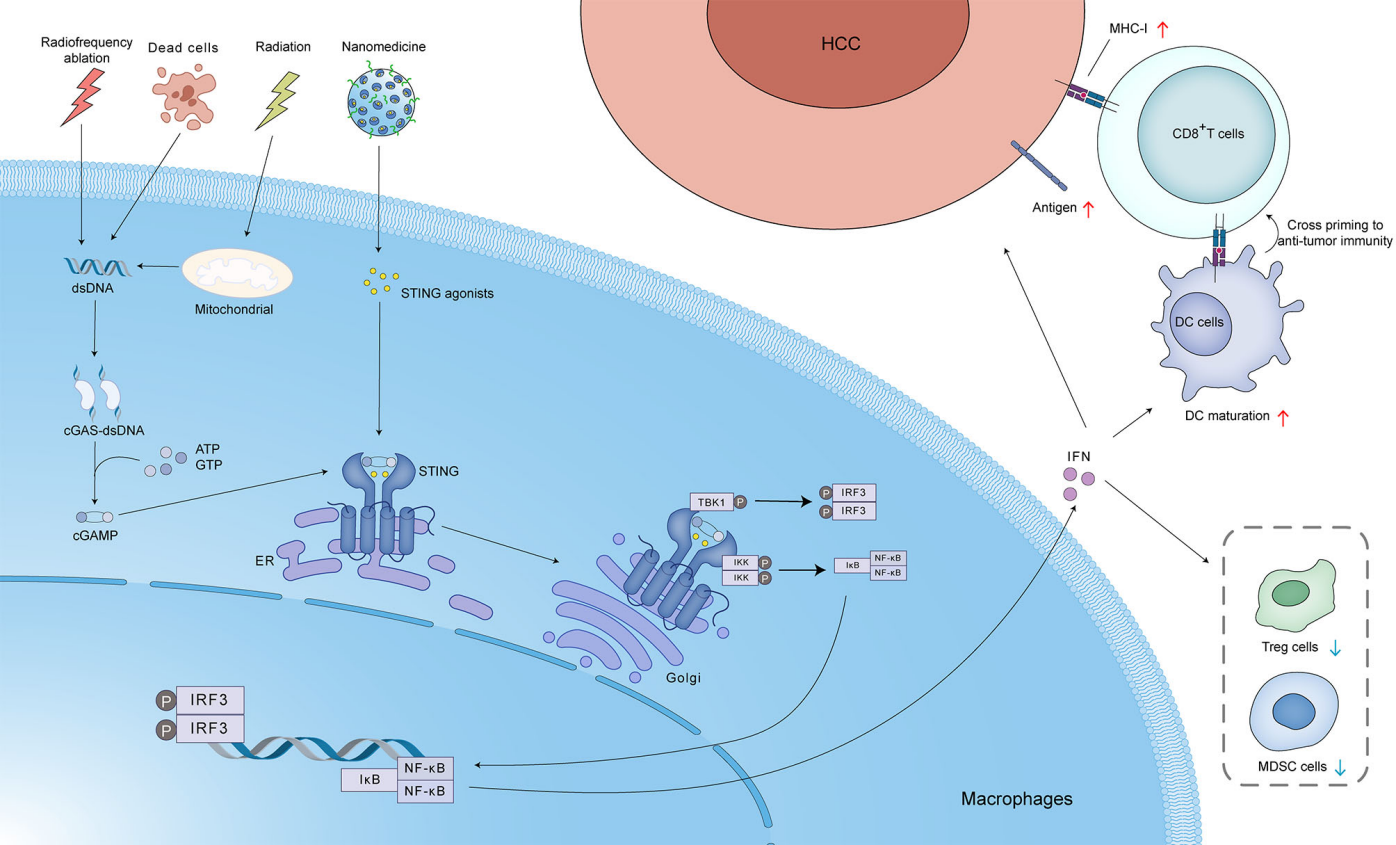
Activation of the cGAS-STING pathway in hepatocellular carcinoma (HCC) by various stimuli, including radiotherapy. Radiofrequency ablation, cell death, radiation, and nanomedicine can induce the release of mitochondrial DNA and/or dsDNA. This dsDNA binds to cGAS, leading to the production of cGAMP. cGAMP acts as a second messenger, activating STING localized in the ER. Activated STING translocates to the Golgi apparatus, where it recruits and activates TBK1 and IKK. TBK1 phosphorylates IRF3, leading to its dimerization and translocation to the nucleus, where it induces the expression of IFN. IKK phosphorylates IκB, leading to its degradation and the release of nuclear factor kappa-light-chain-enhancer of activated B cells (NF-κB). NF-κB also translocates to the nucleus and induces the expression of pro-inflammatory cytokines. IFN released from the tumor cells can act in an autocrine or paracrine manner to upregulate MHC-I expression, promoting antigen presentation to CD8+ T cells. This can lead to cross-priming and activation of anti-tumor immunity. IFN can also promote DC maturation, resulting in enhanced antigen presentation and T cell activation. Additionally, IFN can reduce the number of regulatory T cells (Treg cells) and MDSC cells, which suppress anti-tumor immunity. These processes collectively contribute to the anti-tumor immune response and enhance the efficacy of radiotherapy in HCC.

Stereotactic body radiotherapy (SBRT), a highly promising form of radiotherapy (RT), offers the potential for localized tumor control and enhanced survival rates in patients diagnosed with advanced-stage HCC (52). Ionizing irradiation (IR) impedes tumor growth by triggering DNA damage, including both single-strand breaks (SSBs) and double-strand breaks (DSBs). This damage can trigger the release of dsDNA, leading to cGAS-STING pathway activation and ultimately contributing to the efficacy of radiotherapy against HCC. However, a variety of repair mechanisms, including the prominent pathways of homologous recombination (HR) and non-homologous end joining (NHEJ), can mend DSBs and SSBs. RecQ-Like helicase 4 (RECQL4) is an important DNA damage repair protein that facilitates NHEJ and HR. When HR is deficient, irreparable dsDNA damage accumulates, leading to heightened sensitivity of tumor cells to platinum drugs and poly-ADP-ribose polymerase (PARP) inhibitors. A study conducted by Hong et al. demonstrated that HCC-derived RECQL4 can hinder the anti-tumor immune response through the downregulation of cGAS-STING pathway activation in dendritic cells (DCs). This, in turn, leads to diminished IFN-I synthesis and reduced infiltration of CD8+ T cells into the TME. This process ultimately facilitates tumor evasion of immune surveillance and diminishes the responsiveness of HCC to radiotherapy (53). PARP inhibitors, known for their efficacy in BRCA-mutated cancers, sensitize HCC to radiotherapy by hindering DNA repair. Additionally, they activate the cGAS-STING pathway, promoting cytotoxic T cell recruitment and potentially mitigating immune exhaustion (52), (**Figure 4**).

## 6 The multifaceted role of the cGAS-STING pathway in liver disease: from viral hepatitis to non-alcoholic and alcoholic liver injury

### 6.1 cGAS-STING signaling: a critical pathway in the battle against viral hepatitis

Viral hepatitis, a significant threat to global public health, primarily stems from infections by five distinct hepatotropic viruses: Hepatitis A virus (HAV), Hepatitis B virus (HBV), Hepatitis C virus (HCV), Hepatitis D virus (HDV), and Hepatitis E virus (HEV). Among these viruses, HBV and HCV have the highest fatality rates. In the Asia-Pacific region, particularly, HBV and HCV are the main causes of liver cirrhosis and HCC (54).

#### 6.1.1 HBV infection and cGAS-STING: a complex interplay of evasion and activation

HBV, a hepatotropic DNA virus, is a significant contributor to the global burden of chronic viral hepatitis. The HBV DNA is just 3.2kb, making it the smallest DNA virus discovered to date (55). Chronic HBV infection progression is a major contributing factor to the development of end-stage liver diseases, including cirrhosis and HCC. The transformation of chronic HBV to liver cancer is induced by factors including long-term expression of viral products, such as HBx and LHBs, and HBV DNA’s incorporation into the host’s genetic material.

**Figure 5:**
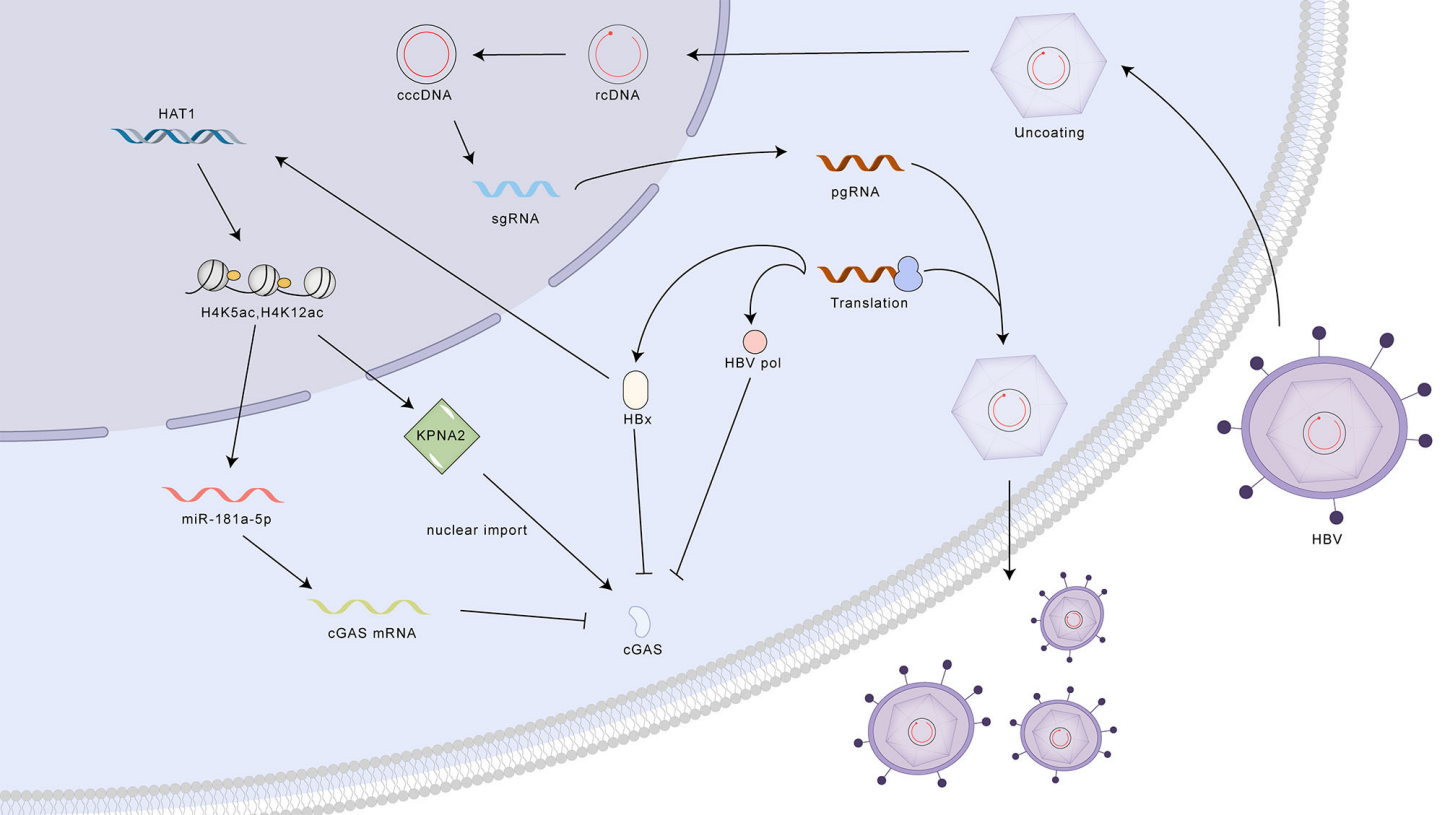
Mechanisms of HBV evasion and activation of the cGAS-STING pathway. HBV employs multiple strategies to evade detection by the cGAS-STING pathway, including nucleocapsid shielding of viral DNA, HBx-mediated cGAS degradation, epigenetic silencing and nuclear relocalization of cGAS, and disruption of STING ubiquitination by HBV polymerase. Despite these evasion tactics, activation of the cGAS-STING pathway through STING agonists can enhance antiviral activity by increasing the production of interferons (IFNs) and interferon-stimulated genes (ISGs). cGAS, cyclic GMP-AMP synthase; STING, stimulator of interferon genes; cccDNA, covalently closed circular DNA; rcDNA, relaxed circular DNA; pgRNA, pregenomic RNA; sgRNA, subgenomic RNA; HBx, HBV X protein; HAT1, histone acetyltransferase 1; miR-181a-5p, microRNA-181a-5p; KPNA2, karyopherin subunit alpha 2; cGAS mRNA, cyclic GMP-AMP synthase messenger RNA.

Despite various treatment methods for HBV, an estimated 290 million people worldwide are chronically infected (56). Multiple hypotheses have been put forth to elucidate the mechanisms by which HBV evades detection by the cGAS-STING pathway: 1. Nucleocapsid Shielding: The HBV nucleocapsid shields viral DNA from cytoplasmic DNA sensors, limiting early immune activation. However, later in infection, nucleocapsid instability may expose DNA, triggering cGAS-STING (57–61). 2. HBx-mediated cGAS Degradation: The viral protein HBx promotes cGAS degradation through ubiquitin-proteasomal pathways and autophagy, suppressing downstream signaling. 3. Epigenetic Silencing and Nuclear Relocalization of cGAS: HBV, via HBx and HAT1, induces epigenetic modifications that upregulate miR-181a-5p, targeting cGAS mRNA and reducing its expression. Additionally, this pathway promotes cGAS nuclear translocation, rendering it ineffective against the virus (62–65). 4. Disruption of STING Ubiquitination: HBV polymerase interacts with STING, disrupting its K63-linked ubiquitination and blocking downstream interferon production and antiviral responses (66) (**Figure 5**).

Despite the strategies used by HBV to evade the cGAS-STING pathway, mounting evidence indicates that stimulating this innate immune response with STING agonists can enhance antiviral activity (60, 67). HBV infection may not effectively trigger the cGAS-STING pathway due to factors like nucleocapsid shielding or inherently low cGAS expression levels in hepatocytes. However, introducing exogenous STING agonists can stimulate this pathway and elicit an antiviral response (60, 61, 67). Stimulating the cGAS-STING pathway has demonstrated efficacy in curtailing HBV replication both *in vitro* and *in vivo* through the reduction of cccDNA levels (61, 67, 68). This antiviral effect is mediated by cGAS-STING-induced cytokines, including IFN-I, IFN-III, and IL-6 (68, 69). Moreover, activating the cGAS-STING pathway leads to increased ISG56 expression, a protein that directly interferes with HBV assembly and RNA synthesis (59). KCs appear to exhibit a dual function in the context of HBV infection. On one hand, they can phagocytose infected cells and initiate apoptosis. On the other hand, the internalization of HBsAg can activate TLR2 on KCs via the cGAS-STING signaling cascade. This activation triggers the release of IL-10, which subsequently suppresses cytotoxic T lymphocyte activity against HBV (70, 71).

The cGAS-STING pathway holds significant potential for the creation of innovative treatments targeting chronic HBV infection. Despite the sophisticated immune evasion tactics of the virus, stimulating this pathway can bolster the innate immune response and potentially control viral replication. cGAMP, a natural STING agonist and intracellular second messenger, can augment HBV-specific immunity when incorporated into vaccine formulations (72). GV1001, a peptide vaccine restricted to MHC class II, triggers the cGAS-STING pathway by promoting mitochondrial dysfunction and the subsequent expulsion of mitochondrial DNA. This mechanism may partially overcome low STING expression in hepatocytes and contribute to overcoming HBV immune evasion (73). Schisandrin C, a compound extracted from the Schisandra plant, has been shown to boost the interaction between TBK1 and STING, leading to increased activation of the cGAS-STING pathway and subsequent upregulation of interferon-beta (IFN-β) and ISGs. Furthermore, this compound has exhibited antiviral activity against the HBV in an HBV mouse model (74). Beyond STING agonists, future research should explore HBV polymerase inhibitors that target the RT/RH domain to prevent disruption of STING ubiquitination. Additionally, epigenetic inhibitors of HAT1 could potentially restore cGAS expression and prevent its nuclear translocation, thereby enhancing cGAS-STING-mediated antiviral responses (75).

#### 6.1.2 HCV and the cGAS-STING pathway: therapeutic opportunities and challenges

Globally, the HCV burden is substantial, with an estimated 58 million individuals experiencing chronic infection, as reported by the World Health Organization (76). A substantial number of individuals with HCV infection progress to a chronic state, potentially leading to severe hepatic complications like cirrhosis and HCC (77). Belonging to the Flaviviridae family, HCV is characterized as a positive-sense, single-stranded RNA virus, possessing a genome that spans roughly 9.6 kilobases. Following receptor-mediated endocytosis, the HCV genome utilizes the internal ribosome entry site (IRES) mechanism to translate a single large polyprotein, which is subsequently processed into individual viral proteins (78, 79).

HCV NS4B, belonging to the group of non-structural proteins, plays a crucial role in the viral life cycle, particularly in replication. This protein has been implicated as a key factor in the innate immune evasion strategies employed by HCV (80–82). Nitta et al. demonstrated that HCV NS4B targets STING, disrupting its interaction with mitochondrial antiviral signaling protein (MAVS) and subsequently inhibiting the activation of the RIG-I-mediated IFN-β signaling pathway (80). Research by Ding et al. revealed that HCV NS4B can disrupt the interaction between STING and its downstream signaling molecule TBK1, thus suppressing the activation of IFN-β (81). Yi et al. showed that the antiviral activity of cGAMP against HCV is genotype-specific. Their findings indicated that cGAMP can effectively curtail the replication of the HCV genotype 1b/Con1 replicon within Huh7.5 cells. This inhibition occurs by activating STING, consequently triggering the release of INF-Is and inflammatory cytokines (82).

While direct-acting antiviral drugs (DAAs) have achieved cure rates exceeding 90% for HCV, challenges such as high cost, drug-resistant strains, and reinfection remain significant obstacles to global eradication (83–86). Prophylactic vaccination, capable of eliciting both antibody and cell-mediated immunity, is viewed as a crucial approach for establishing durable HCV control and potentially achieving global elimination (87–89). Landi et al. compared the immunogenicity of an HCV rE1E2 vaccine adjuvanted with the STING agonist c-di-AMP to that of vaccines formulated with established adjuvants like MF59 and aluminum/MPL. Their findings in mice demonstrated that c-di-AMP, when used as a vaccine adjuvant, elicited superior humoral and cellular immune responses compared to the other adjuvants (90). The results indicate that activating the STING pathway during vaccination may significantly improve the immune response against HCV, thereby paving the way for the creation of more potent preventive strategies against the virus.

### 6.2 cGAS-STING activation in metabolic dysfunction-associated steatotic liver disease: driving inflammation and disease progression

Metabolic dysfunction-associated steatotic liver disease (MASLD), previously termed non-alcoholic fatty liver disease (NAFLD), is characterized by the buildup of excess lipids within liver cells. This accumulation is primarily fueled by metabolic imbalances and exhibits a strong correlation with both insulin resistance and the genetic makeup of individuals (91). Hepatic steatosis is the hallmark of MASLD. While simple steatosis is generally considered benign, the presence of significant inflammation and hepatocellular injury marks the progression to metabolic dysfunction-associated steatohepatitis (MASH, formerly known as NASH) (92, 93). MASH has the potential to advance to cirrhosis, HCC, and ultimately culminate in hepatic decompensation (94–97).

**Figure 6:**
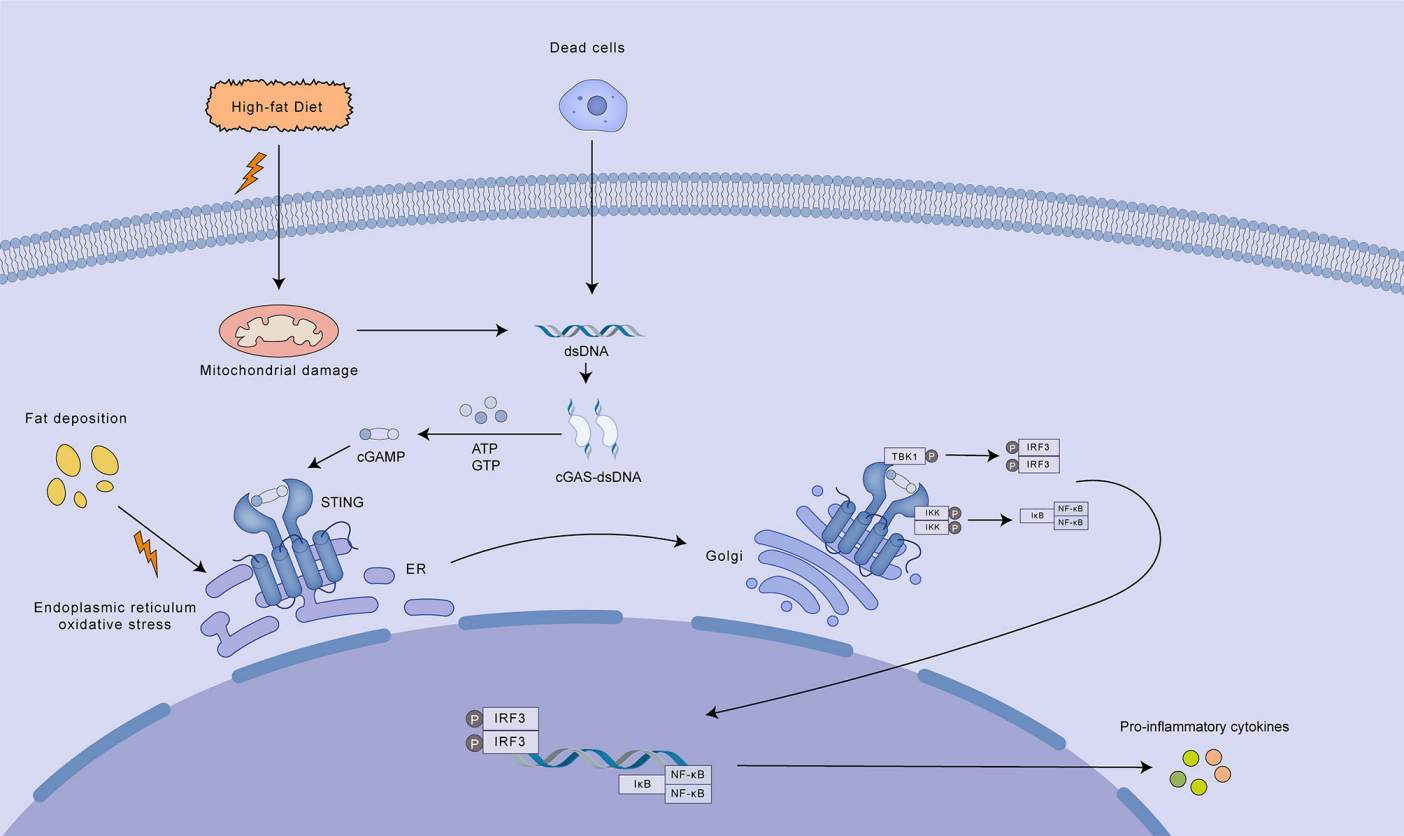
Mechanisms of cGAS-STING pathway activation in MASLD/MASH. A high-fat diet contributes to mitochondrial damage and ER oxidative stress, leading to the release of mtDNA and other damage-associated molecular patterns (DAMPs). Cytosolic mtDNA activates the cGAS-STING pathway, resulting in the production of cGAMP. cGAMP acts as a second messenger, binding to and activating STING located on the ER membrane. Activated STING translocates to the Golgi apparatus where it recruits and activates TBK1. TBK1 subsequently phosphorylates IRF3, promoting its dimerization and nuclear translocation. Nuclear IRF3, along with NF-κB, induces the transcription of pro-inflammatory cytokines, contributing to the inflammatory response characteristic of MASLD/MASH. Additionally, dead cells can release dsDNA, which further activates the cGAS-STING pathway, amplifying the inflammatory response.

Several mechanisms are believed to contribute to cGAS-STING pathway stimulation in the context of MASLD/MASH: 1. Mitochondrial dysfunction, a hallmark of MASLD/MASH, can result in the accumulation of mtDNA, which in turn triggers the activation of the cGAS-STING pathway (98). 2. Alternatively, the engulfment of apoptotic or necrotic hepatocytes by liver macrophages can liberate hepatocyte-derived nucleic acids, notably DNA, which subsequently triggers cGAS-STING signaling in the macrophage cytosol (99). 3. It has also been proposed that excessive hepatic lipid accumulation in MASLD patients can lead to ER stress, subsequently activating the STING-TBK1 pathway and promoting inflammation (100) (**Figure 6**).

The cGAS-STING pathway, predominantly driven by liver-resident macrophages (especially KCs), plays a crucial role in the pathogenesis and advancement of MASLD. Yu’s study demonstrated that STING deficiency attenuated liver steatosis, fibrosis, and inflammation in a mouse model of MASLD. Subsequent investigations revealed that STING deficiency suppressed mtDNA-induced NF-κB activation, as well as the production of TNF-α and IL-6 in KCs. TNF-α significantly exacerbates hepatic steatosis, while IL-6 potently activates lipogenic factors in hepatocytes. Collectively, these findings suggest that STING mediates liver steatosis, fibrosis, and inflammation through promoting NF-κB activation and subsequent TNF-α and IL-6 production in KCs (101). Luo X et al. investigated the role of STING in MASLD/MASH using a high-fat diet (HFD)-induced mouse model. Loss-of-function studies demonstrated that mice with STING specifically deleted in hepatic macrophages exhibited reduced liver steatosis and inflammation compared to mice with intact STING expression. Conversely, gain-of-function studies showed that mice with STING expression restricted to hepatic macrophages developed more severe liver steatosis and inflammation compared to mice with global STING deletion (102). Examination of hepatic tissue from a cohort of 98 individuals diagnosed with MASLD revealed a positive association between STING levels in KCs and other hepatic macrophages, and the severity of both inflammation and fibrosis within the liver (103). Collectively, these findings suggest that STING plays a detrimental role in the pathogenesis of MASLD/MASH, with cGAS-STING pathway activation primarily occurring in non-parenchymal cells, particularly hepatic macrophages, contributing to their pro-inflammatory effects.

Given that sustained stimulation of the cGAS-STING pathway is implicated in the progression of MASLD, suppressing this pathway presents a potential therapeutic avenue for mitigating hepatic inflammation. Consequently, considerable research endeavors have been directed towards the development of STING inhibitors. STING inhibitors can be broadly classified as covalent or non-covalent inhibitors. Covalent inhibitors irreversibly bind to STING, leading to pathway inactivation, while non-covalent inhibitors competitively inhibit 2’,3’-cGAMP binding, thereby modulating STING pathway activation (104–108). Remdesivir (RDV) has been shown to inhibit MASLD progression by ameliorating dyslipidemia and inflammatory responses, with its regulatory effects attributed, in part, to the blockade of the STING signaling pathway (109). Therefore, RDV may represent a potential therapeutic option for MASLD. Cinnamaldehyde and glycyrrhizic acid, key components of Linggui Zhugan Decoction (LGZG), have been shown to ameliorate HFD-induced hepatic steatosis by suppressing STING-TBK1 pathway activation in KCs and reducing the release of inflammatory cytokines such as IFN-β and TNF-α (110). Sorafenib has been demonstrated to attenuate MASLD progression by inducing autophagic degradation of cGAS, STING, TBK1, and IRF3. Furthermore, low-dose sorafenib has been shown to inhibit MASH progression in both murine and primate models (111, 112). Since sorafenib is a multi-kinase inhibitor with a broad range of targets, its effects on MASLD/MASH may extend beyond STING inhibition and require further investigation.

### 6.3 cGAS-STING signaling and the pathogenesis of alcoholic liver disease (ALD): fueling the inflammatory fire

Alcoholic liver disease (ALD) manifests as a range of hepatic injuries resulting from chronic and high levels of alcohol intake. These injuries can progress from the initial stage of fatty liver accumulation (hepatic steatosis) to more severe conditions, including inflammation and fat accumulation (steatohepatitis), liver scarring (fibrosis), advanced scarring (cirrhosis), and eventually, HCC.

**Figure 7:**
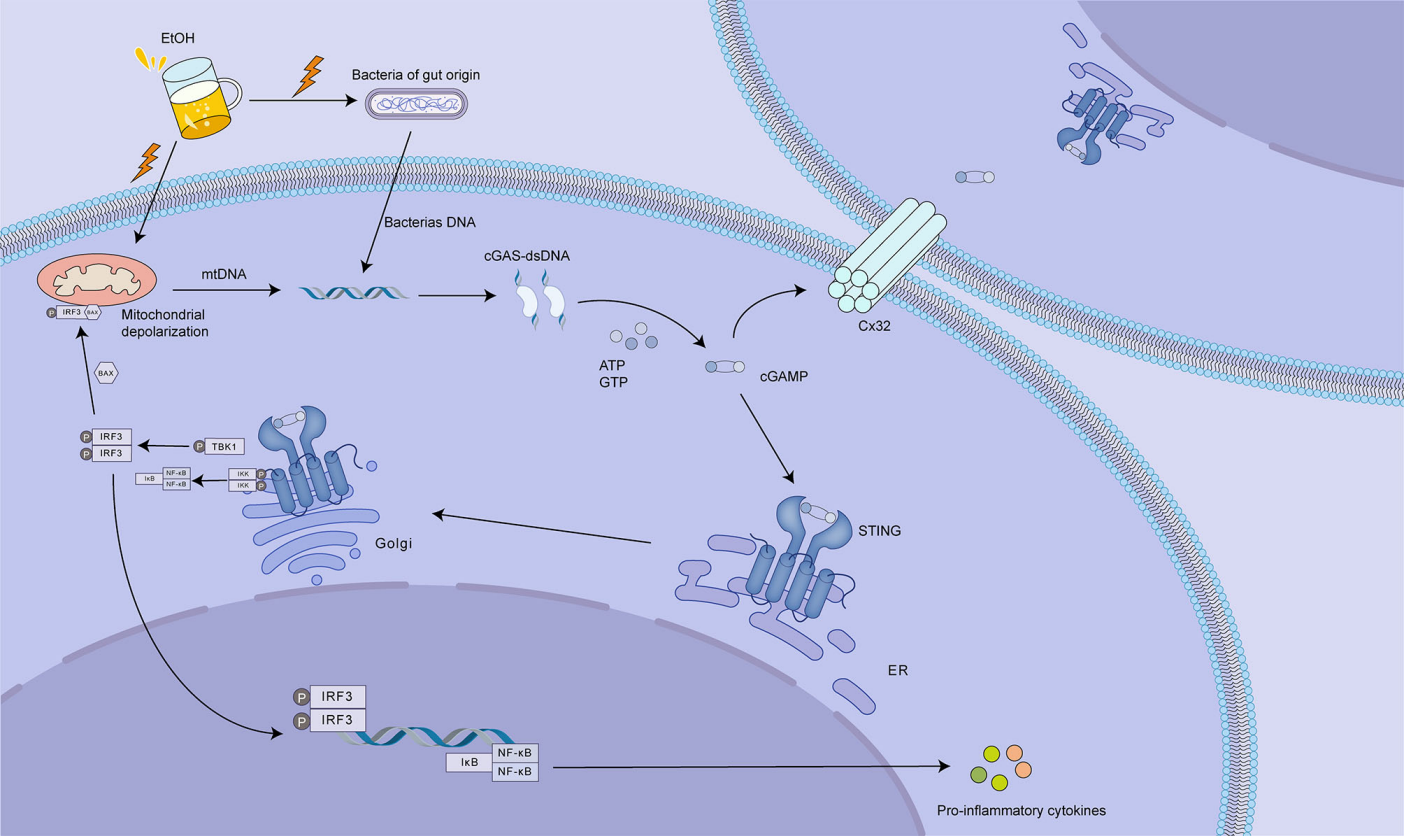
The role of cGAS-STING signaling in alcoholic liver disease (ALD). Excessive alcohol consumption (EtOH) disrupts gut barrier integrity, leading to increased translocation of gut-derived bacteria and bacterial DNA. This, coupled with ethanol-induced mitochondrial damage and release of mtDNA, activates the cGAS-STING pathway. cGAS binds to bacterial or mtDNA, producing cGAMP. cGAMP then activates STING, located on the ER, triggering downstream signaling that culminates in IRF3 phosphorylation and activation. Activated IRF3 translocates to the nucleus, promoting the expression of pro-inflammatory cytokines. Notably, cGAMP can also be transferred to neighboring hepatocytes via connexin 32 (Cx32) gap junctions, amplifying the inflammatory response. This intercellular signaling contributes to hepatocyte death and the progression of ALD. Targeting cGAS, STING, or Cx32 could offer therapeutic strategies to mitigate liver injury in ALD by dampening the inflammatory cascade.

Activation of the cGAS-STING pathway contributes to the pathogenesis and progression of ALD, potentially exacerbating inflammatory damage through its interaction with the gap junction protein connexin 32 (Cx32) (113). Excessive alcohol consumption can induce mitochondrial damage in hepatocytes, leading to the release of mtDNA. Additionally, damaged or apoptotic hepatocytes release damage-associated molecular patterns (DAMPs), while gut dysbiosis associated with ALD can lead to increased translocation of pathogen-associated molecular patterns (PAMPs), such as lipopolysaccharide (LPS) (114). These DAMPs and PAMPs activate the cGAS-STING pathway, culminating in the production of IRF3 (113, 115). Beyond its role in promoting IFN-I expression, IRF3 has been shown to engage with BAX, a protein known to induce apoptosis, thus potentially contributing to hepatocyte death and the ensuing inflammatory response (116). An investigation examining hepatic tissue from 51 individuals with ALD revealed a direct association between the levels of IRF3 and associated gene expression and disease severity (113). Further research has revealed that gap junctions composed of Cx32 between hepatocytes facilitate the rapid intercellular transmission of signals. These gap junctions facilitate the intercellular propagation of cGAMP emanating from damaged hepatocytes, thereby promoting a broader activation of IRF3 and potentially accelerating the progression of ALD (117). Inhibition of Cx32 function, akin to a firewall, can effectively prevent the spread of inflammatory signals between hepatocytes, thereby attenuating the progression of ALD (113) (**Figure 7**).

The stimulation of IRF3 can play a role in ethanol-induced hepatocyte death, initiating a substantial subsequent inflammatory cascade and contributing to the development of alcoholic liver disease. Consequently, inhibiting cGAS and Cx32 activity could hold potential as therapeutic interventions to lessen hepatic injury in ALD.

## 7 Conclusion

The cGAS-STING axis has emerged as a pivotal mechanism for sensing cytoplasmic DNA, with a central function in orchestrating both the pro-inflammatory and anti-tumorigenic facets of the INF-I response. The cGAS-STING pathway constitutes a key element of hepatic innate immunity. While hepatocytes themselves lack significant STING expression, other liver resident cells, such as liver sinusoidal endothelial cells and KCs, express STING and contribute to the activation of this pathway in response to pathogens or cellular damage. Indeed, the cGAS-STING pathway displays a multifaceted, Janus-faced influence on hepatocellular carcinoma (HCC) pathogenesis. In the context of chronic liver diseases, including MASLD and alcoholic hepatitis, persistent heightened activity of the cGAS-STING cascade can exacerbate hepatic inflammation, potentially accelerating disease advancement and even fostering HCC development. Conversely, in the context of established HCC, short-term stimulation of the cGAS-STING cascade has demonstrated the ability to diminish tumor load in advanced HCC and reconfigure the TME, thereby augmenting anti-tumor immunity. In viral hepatitis, activation of the cGAS-STING pathway inhibits viral propagation and virion formation, thereby enhancing the immune response mounted by the host against the virus.

cGAS-STING represents a vital element within the innate immune response, functioning as a detector of both intracellular and foreign DNA. Stimulation of the STING pathway has the capacity to bolster host defenses against infectious agents, facilitate tumor suppression, and potentially provide clinical advantages for individuals battling viral hepatitis and advanced-stage HCC. However, chronic or excessive STING activation can also disrupt hepatic metabolic homeostasis and contribute to sterile inflammation and immune dysregulation, potentially exacerbating the progression of inflammatory liver diseases. Therefore, the therapeutic application of STING agonists in the context of liver disease, particularly during the progression from chronic liver disease to HCC, requires careful consideration and a thorough understanding of its context-dependent effects. Despite its complex and context-dependent roles in liver disease, STING remains a promising therapeutic target, particularly for HCC and viral hepatitis.

Although the cGAS-STING axis plays a critical role in the pathogenesis of numerous hepatic diseases, further clinical investigation is warranted to assess its therapeutic potential. Future research should focus on addressing key challenges, including targeted delivery of STING agonists to tumor tissues, precise modulation of STING activity at different stages of disease progression, cell-type specific targeting, and strategies for fine-tuning STING activation levels to maximize therapeutic benefit and minimize potential adverse effects. Addressing the challenge of delivering tumor- or liver-specific STING agonists is paramount, as the inability to accurately target neoplastic tissue hinders subsequent regulatory interventions. The next critical step involves establishing stage-specific STING modulation strategies for liver disease, necessitating the precise determination of optimal time points for STING pathway activation or suppression to maximize therapeutic benefit. Subsequently, achieving cell type-specific STING interventions is crucial, requiring the development of microenvironment-tailored STING modulation strategies that target distinct cellular populations within the diseased liver, while concurrently preserving normal hepatocyte function. Ultimately, optimization of dynamic STING activation hinges on the creation of microenvironment-responsive, “intelligent” STING modulators capable of precisely controlling local drug release in a spatiotemporal manner, thus mitigating the risks of both tissue damage from overactivation and therapeutic resistance resulting from inadequate stimulation.

Ultimately, the cGAS-STING pathway embodies both a beacon of hope and a cautionary tale in liver disease therapeutics. Its dual roles demand a nuanced approach—harnessing its protective potential while mitigating its inflammatory excesses. By bridging mechanistic insights with technological innovation, we can unlock its full potential to transform the landscape of hepatic disease treatment, paving the way for personalized and precision medicine in hepatology.
